# The effect of a savings intervention on women’s intimate partner violence victimization: heterogeneous findings from a randomized controlled trial in Colombia

**DOI:** 10.1186/s12905-019-0717-2

**Published:** 2019-01-25

**Authors:** Margaret E. Tankard, Elizabeth Levy Paluck, Deborah A. Prentice

**Affiliations:** Department of Psychology, Princeton University, Princeton, NJ 08540 USA

**Keywords:** Intimate partner violence, Economic empowerment, Financial wellbeing, Mental health, Randomized controlled trial

## Abstract

**Background:**

Women’s economic empowerment has long been assumed to lead to their social empowerment, but systematic tests of this relationship have only recently begun to appear in the literature. Theory predicts that control over resources, as through a savings account, may increase women’s negotiating power and self-efficacy. In this way, “economic empowerment” may lead to “social empowerment,” and have related benefits such as helping to reduce risk of intimate partner violence (IPV). The current study tests effects of an economic empowerment intervention on women’s social empowerment, IPV victimization, and health.

**Methods:**

We conducted an 18-month randomized controlled trial among 1800 urban poor women in Colombia between 2013 and 2015. The trial tested the impact of a savings account offer bundled with health services (vs. health services alone) on social empowerment outcomes, IPV victimization, and health.

**Results:**

The bundled savings treatment did not have average effects on most outcomes, although it produced a small significant increase in financial participation and decrease in symptoms of depression. Treatment effects on perceived norms, decision-making patterns, self-reported IPV victimization, and health depended on whether women’s partnerships were free of violence when they entered the trial; specifically, women in nonviolent partnerships at baseline showed more positive effects of the intervention.

**Conclusions:**

Although bundling economic empowerment interventions with support features has been shown to empower poor women, this trial found that a bundled treatment did not on average improve most social and health outcomes of poor women experiencing IPV.

**Trial registration:**

Registered retrospectively, prior to realization of outcomes, 5/29/14: Evidence in Governance and Politics #20140529AA.

**Electronic supplementary material:**

The online version of this article (10.1186/s12905-019-0717-2) contains supplementary material, which is available to authorized users.

## Background

Women’s economic empowerment has long been assumed to lead to their social empowerment, including their autonomy and wellbeing within their families and societies. However, systematic tests of this relationship have only recently begun to appear in the literature [1]. Even fewer trials have specifically examined whether economic empowerment reduces intimate partner violence (IPV), and these trials have yielded inconsistent results [2–5].

For example, recent trials provide some evidence that economic programs introducing an influx of new resources to a household, such as cash transfers, may reduce IPV as a function of lowering household stress and conflict [6, 7]. Yet new household resources may not be enough to directly alter women’s economic or social empowerment. Empowerment has been defined as the “expansion of freedom of choice and action to shape one’s life” through “control over resources and decisions” in the economic and social domains (p. 4) [8]. New resources in a household may not remain under women’s control, and may not change patterns of status and influence more generally within a household.

By contrast, savings accounts allow women to save for their goals, and to protect their money from economic shocks and from family members and others who ask them for money [9]. Strong and accumulated evidence demonstrates that savings accounts empower all types of women economically, in the form of increased control over resources [1]. Little is known about whether savings accounts also empower women socially and psychologically, in the form of increased actual control over household decisions and in the form of perceived control and self-efficacy such as confidence and felt independence [10]. Relatedly, it is unknown whether this kind of social empowerment has the effect of preventing or reducing IPV [2, 5]. Ecological theories of IPV, which analyze influences from individual to societal, hold that power dynamics at micro and macro levels can render individual women vulnerable to IPV [11]. Theories focused on women’s empowerment have in turn posited that increasing women’s power at the individual level will increase their safety and health [12]. More specifically, theory predicts that control over resources, as through a savings account, may increase women’s negotiating power and self-efficacy, leading to improved treatment by their partner or a better ability to leave the relationship if that is what women desire [4]. Of course, women’s empowerment is not the only possible way or even the most normatively desirable way to reduce IPV; however, it may be an effective way to reduce IPV. The present research targeted savings accounts to test the links among economic empowerment, social empowerment, and reduced IPV.

In an 18-month randomized controlled trial, we examined the effects of savings accounts on the social and health outcomes of a random sample of 1800 poor women in urban Colombia. We chose an urban rather than rural setting to extend previous research on empowerment and IPV, which has focused on rural settings [1, 13].

Colombia has made substantial progress toward establishing laws protecting women’s rights, including the landmark Law 1257, adopted in 2008, which issued regulations to prevent and punish violence and discrimination against women [14]. However, IPV remains a major issue. An estimated 32% of ever-partnered Colombian women ages 13–49 have experienced physical violence from their current or last intimate partner [15], and Colombia had the second highest 12-month prevalence rate of physical partner violence in a comparative analysis of 12 Latin American countries [16].

Nationally, Colombia has ranked in the top quartile on the World Economic Forum’s global index of gender parity in economic participation and opportunity [17]. However, because participants in the present study were poor,^1^ we bundled the savings accounts with health services and intervention-related support. Previous research has shown that, to improve their economic standing, poor women may require an economic intervention to be bundled with support in areas such as life skills and health [1]. In addition, the literature on IPV reduction sometimes finds that the effectiveness of interventions depends on characteristics of women and their partners, such as relative economic position and initial relationship quality [3–5]. Engaging a random sample of women, who varied in relationship quality, income sources, and other dimensions of vulnerability, enabled us to investigate these heterogeneous effects.

## Methods

This study was undertaken in accordance with Consolidated Standards of Reporting Trials (CONSORT) guidelines.

### Participants

We recruited sample of 1800 female participants across four low-income neighborhoods in Cali, Palmira, and Buenaventura, Colombia (see Table 1). Each site contained a branch of the project’s partnering bank and health clinic. Eligibility requirements for participation in the study included being a woman of age 18–55 who has a male partner (not necessarily cohabiting), who had not used a formal or informal savings service or any service of the partnering bank within the past 12 months, and who indicated preliminary interest in opening a savings account (see Additional file 1 for additional detail).

**Table 1:** Baseline demographics by condition

	Control Group		Treatment Group		Difference
	*M*	*SD*	*M*	*SD*	*p*
Site: Poblado (y/n)	0.49	0.50	0.48	0.50	0.741
Site: Versalles (y/n)	0.05	0.22	0.06	0.24	0.317
Site: Palmira (y/n)	0.21	0.41	0.20	0.40	0.617
Site: Buenaventura (y/n)	0.26	0.44	0.27	0.44	0.617
Age at recruitment	32.92	10.27	33.69	10.27	0.171
Partnership is marriage or civil union (y/n)	0.78	0.41	0.78	0.42	1.000
Cohabiting with partner (y/n)	0.72	0.45	0.73	0.44	0.617
Has children (y/n)	0.84	0.37	0.84	0.37	1.000
Number of household residents	4.68	1.76	4.57	1.91	0.271
Number of recent stressful events (0–7 count)	1.06	0.97	1.09	0.97	0.549
Frequency of talking to neighbors (1–6)	3.13	1.74	3.03	1.66	0.267
Ethnicity: Afrocolombiana (y/n)	0.41	0.49	0.39	0.49	0.503
Ethnicity: Blanca (y/n)	0.23	0.42	0.21	0.41	0.317
Ethnicity: Mestiza (y/n)	0.26	0.44	0.30	0.46	0.046
Ethnicity: Mulata (y/n)	0.06	0.25	0.06	0.24	1.000
Neighborhood SES level (1–6)	1.99	0.64	1.96	0.65	0.317
Working (y/n)	0.42	0.49	0.43	0.49	0.741
Subjective SES (1–10)	4.49	2.12	4.72	2.18	0.055
Log-transformed income	12.54	1.63	12.71	1.11	0.034
Current formal financial services (y/n)	0.13	0.34	0.14	0.35	0.617
Putting money aside past 6 months (y/n)	0.39	0.49	0.42	0.49	0.317
Saving for purpose past 6 months (y/n)	0.39	0.49	0.41	0.49	0.503
Identification with Colombians (1–4)	3.66	0.45	3.67	0.47	0.741
Identification with women in community (1–4)	3.13	0.83	3.12	0.85	0.842
Education: college started or completed (y/n)	0.24	0.43	0.23	0.42	0.617
Education: none through middle school (y/n)	0.76	0.43	0.77	0.42	0.617
Sexually active (y/n)	0.92	0.27	0.93	0.25	0.317
Any IPV (y/n)	0.44	0.50	0.42	0.49	0.503
IPV index (0–11 count)	1.14	1.89	1.12	1.94	0.920
Financial IPV index (0–2 count)	0.20	0.47	0.23	0.51	0.317
Emotional IPV index (0–5 count)	0.71	1.17	0.65	1.13	0.407
Physical IPV index (0–2 count)	0.17	0.5	0.17	0.49	0.741
Sexual IPV index (0–2 count)	0.06	0.3	0.07	0.32	0.617
Enumerator rating of participant comfort (1–7)	6.40	0.76	6.37	0.83	0.453
Enumerator rating of participant honesty (1–7)	6.45	0.74	6.46	0.71	0.617

We do not find differences by condition for most baseline demographics. Where we find a difference by Mestiza ethnicity, it is not paralleled by differences in other ethnicities. Where we find a difference by log-transformed income, paralleled by a marginally significant difference in subjective socioeconomic status (SES), we control for SES in our analyses. Numeric ranges in parentheses refer to the response scale; y/n indicates a binary yes/no response scale

### Procedure

We surveyed participants at the start of the project (beginning June 2013) and two more times (9 and 18 months later). A team of female enumerators employed by a Colombian survey research firm recruited participants using a random walk method that randomly determined the number of residences to skip (2 or 3) before approaching the next household. After ensuring that a prospective participant passed the series of eligibility requirements, they invited her to participate in a project called Proyecto Crecer (Project Grow), presented as a set of social programs related to health and wellbeing.

**Fig. 1:**
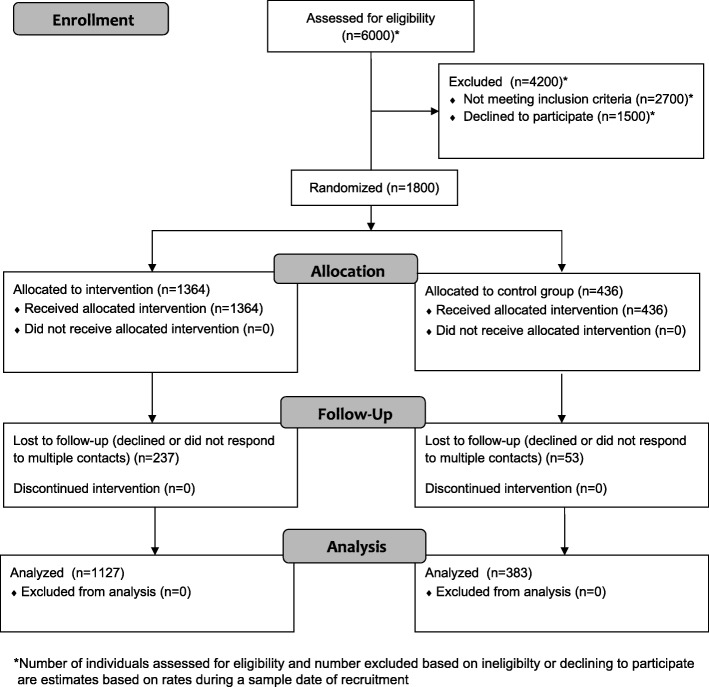
CONSORT flow diagram of participant enrollment

Participants completed a baseline survey verbally with the enumerator in their home, and then were randomly assigned to receive either a savings account bundled with health services (*N* = 1364) or just health services (*N* = 436), as shown in Fig. 1 (see Additional file 1 for further information regarding sample size). At the end of the baseline survey, participants were given a voucher for three free health checkups at a health clinic, providing access to a pre-designated set of services including a medical checkup, serology, and a family planning consultation. Personal phone calls and SMS (short message service) messages from the survey firm reminded participants to attend their checkups. Participants were encouraged to attend close to the time of each of the three survey waves, but were able to schedule a checkup at any point during the project.

### Intervention

The bundled treatment combined the free health checkups with a free, no-fees, personal savings account. Participants could open the savings account at a local bank branch in their neighborhood (see Additional file 1 for details). The savings account was enhanced in two ways: An initial deposit of 10,000 pesos (~$5 U.S. dollars; USD) was funded by the project for any participant who wanted to open an account, and subsequent deposits to the account were matched by project funds at a rate of 1/3 up to a limit. During the recruitment phase, enumerators used a visual aid to explain how the savings account worked and then asked if the participant wanted to open it. If she did, enumerators explained that she only needed to arrive at the bank, sign, and give her fingerprint, as the initial deposit would be provided by the project. We used behavioral strategies to support participants’ efforts to open an account, including a map with directions to the bank, and text message encouragements, both of which were designed by local project staff. Other incentives, such as small lotteries held at the bank for anyone opening or using the account, were developed as the project unfolded to encourage continued usage and account opening.

### Data collection

We attempted to survey the full original sample in the follow-up surveys, which were scheduled by phone and took place at participants’ home with an enumerator, as in the baseline survey.^2^ Participants were compensated with a gift certificate with a value of 10,000 Colombian pesos (equivalent to $5 USD at the start of the project) for completing the baseline survey, a value of 15,000 pesos for completing the 9-month survey, and a value of 30,000 pesos for completing the 18-month survey. We also collected data recorded during women’s health services (an additional informed consent procedure was implemented at the health services for use of the health data in the study).

### Measures

We recorded outcome measures through the surveys and health checkup data, and we additionally partnered with the bank to track all women’s banking activity. Below, we describe survey indexes compiled from individual questions, followed by health checkup measurements. See Additional file 1 for additional detail regarding measures.

#### Formal banking experience

Enumerators asked participants a series of items regarding participants’ trust in banks, negative perceptions of banks (reverse-scored), perceived difficulty of going to the bank (reverse-scored), and self-reported formal saving behavior in the last six months. We used these items to create a standardized weighted index (see Analysis section for details) of positive perceptions of and engagement with formal banking.

#### Economic status

Survey items regarding participants’ monthly income, subjective socioeconomic status, and work status, were combined into a standardized weighted index of economic status.

#### Confidence

Survey items regarding participants’ feelings of self-efficacy as related to accessing resources under challenging circumstances, their self-esteem, and their optimism regarding the future were combined into a standardized weighted index of confidence.

#### Attitudes of social empowerment

Survey items regarding participants’ feelings about and self-reported behavior of following a partner’s wishes (reverse-scored), their personal justification for their partner’s use of IPV in different situations (reverse-scored), and personal belief that women should tolerate IPV to keep the family together (reverse-scored) were combined into a standardized weighted index of attitudes of social empowerment.

#### Perceived norms of social empowerment

We asked a series of survey items regarding participants’ perceptions of *other* women in the community: their feelings about and self-reported behavior of following a partner’s wishes (reverse-scored), their personal justification for their partner’s use of IPV in different situations (reverse-scored), and personal belief that women should tolerate IPV to keep the family together (reverse-scored), and their perception that women are becoming more supportive (vs. not changing or becoming less supportive) of women having control over their finances and lives. We combined them into a standardized weighted index of perceived norms of social empowerment.

#### Ending of relationship

In follow up surveys, participants were asked if they were in the same relationship that they were in when they were first surveyed in the project.

#### Independent decision-making

We derived a standardized weighted index of independent (vs. collaborative) decision-making from several types of survey questions. First, participants responded to questions about how decisions are made in their household [10]. For five topics, participants were asked who makes most of the decisions (themselves, their partner, or both) from a list (answering all that applied): what to buy at the market, purchase of expensive items, recreational use of money, schooling of children, and visits to parents or other family members. We calculated the proportion of decisions made independently, as opposed to jointly or by the partner. Participants were also asked whether or not they initiate discussions about each topic, and we calculated the proportion of decisions for which participants initiate discussions.

Second, at the end of the survey, we presented participants with a gift certificate as compensation for their time responding. They were asked whether or not they would tell their partner about the gift certificate (not telling him was coded as greater independent decision-making). They were also asked who would decide how to use the gift certificate (for themselves, their partner, or for both); we coded whether or not participants would decide independently.

Third, participants responded to two independent items regarding their partner’s knowledge of how much money they have (reverse-scored) and whether they talk to their partner about this project (reverse-scored), respectively.

Research has thus far not established the precise relationship between the form of decision-making and “social empowerment”, particularly how independent and collaborative decision-making each correspond to patterns of influence and closeness in intimate relationships. In the present findings, we interpret the independent decision-making index not as an inherently positive outcome, but rather as an indicator of the extent to which the respondent is making decisions on her own, for better or worse.

#### Intimate partner violence (IPV) victimization

To measure IPV victimization, we used an unweighted index for greater interpretability of effects (the results are consistent when using a weighted index, and across the different subtypes of IPV and a scale of relationship satisfaction). Enumerators showed participants a response card so that they could point to their answer rather than speak aloud, for increased confidentiality in the home. Participants were asked whether or not, in the past six months, their partner had engaged in each of 11 different violent and controlling behaviors [18]. These behaviors were related to financial violence (e.g., “taken your earnings or savings against your will”), emotional violence (e.g., “insulted you or made you feel bad about yourself”), physical violence (e.g., “thrown something at you, slapped you, pushed you, or crushed you”), and sexual violence (e.g., “physically forced you to have sexual relations against your will”). We calculated the total number of IPV behaviors reported across all 11 items (0–11 scale).

#### Health checkup measures

At the health checkups, the providers recorded whether participants accepted an offer to receive a test for sexually transmitted infections (STIs) and an offer to receive a family planning consultation. They used abbreviated self-report scales of stress, depression, and anxiety to assess symptoms of psychological distress (0–4 scale), and recorded blood pressure. They also recorded whether they identified signs of physical injury on the participants’ body (e.g., bruises, cuts), whether the participant self-reported experiencing different forms of emotional, physical, or sexual violence when asked (0–7 scale), and the frequency with which participants self-reported experiencing violence (0–4 scale).

### Analysis

We used linear regression to analyze the effect of the bundled savings treatment (vs health services alone) on 18-month survey measure indexes. For weighted indices, items were combined using principal components analysis, and standardized based on the control group of the respective wave. As an additional strategy to account for multiple comparisons, we used seemingly unrelated regression (SUR) to estimate the system of equations for each family of measures, conducting a Wald test of the joint significance of coefficients of interest.

We also analyzed the treatment effect on averaged post-treatment data recorded during women’s health services. We computed each participant’s average response value for each outcome measure across the number of checkups that the participant attended. This analytic strategy offers the strength of including all subjects who attended any checkups and not overweighting participants who were particularly interested or uninterested in attending.

We conducted intention-to-treat analyses, analyzing all responses recorded based on savings treatment assignment, regardless of whether a participant took up the savings account offer. We estimated robust standard errors and controlled for participants’ socioeconomic status (an index based on neighborhood modal social class, income, subjective socioeconomic status (SES), work status, and education level), life stage (an index based on age, having children, and being married or in a civil union), and project site. We first tested the effect of the savings treatment on each dependent measure. We then tested whether the effects of financial treatment differed depending on participants’ baseline self-reports of IPV victimization. Specifically, we interacted condition assignment with a dummy variable indicating whether a participant self-reported experiencing violence from her partner in any of the 11 listed violent behaviors in the baseline survey. At baseline, 43% of participants (768 out of 1800) reported experiencing any of the 11 listed violent behaviors.

## Results

The retention rate in the 18-month survey was 83% in the treatment group and 88% in the control group (see Fig. 1), a difference that was significant (95% confidence interval [CI] = − 0.09, − 0.01). The control group was also more likely (78%) than the treatment group (61%) to attend at least one health checkup, a difference that was significant (95% CI = − 0.21, − 0.13; qualitative work suggested that participants in the control group viewed the health services as the central element of the project). Among the 1364 participants assigned to treatment, 690 (49%) opened an account, 455 (33%) made at least one deposit, and 286 (21%) made at least one withdrawal. Among the 455 who made a deposit, the median total deposited across the project was 180,000 Colombian pesos (equivalent to $95 USD at the start of the project).

### Average effects

Compared to health services alone, the bundled savings treatment encouraged saving and increased women’s formal financial participation (treatment coefficient: B = 0.42, 95% CI = 0.31, 0.53). It promoted more independent (vs collaborative) decision-making in relationships (B = 0.11, 95% CI = 0.00, 0.21), although this effect was not robust. The bundled treatment also decreased self-reported symptoms of depression at the health services (B = − 0.10, 95% CI = − 0.18, − 0.02).

Notably, there were no overall effects of the bundled savings treatment on financial wellbeing, on other aspects of mental health, or on a range of psychological indicators of social empowerment. We measured multiple aspects of social empowerment including confidence, women’s personal attitudes regarding their partnership, and perceived norms of women’s social empowerment, which represents a departure from the previous literature. There were no average effects on participants’ relationship status, self-reported IPV victimization, or physical injuries observed at the health service. See Table 2 for all average treatment effects.

**Table 2:** Average treatment effects

Dependent Measure	Average Treatment Effect					
	B	[95% CI	95% CI]	p	N	Joint p
Survey Measures						
Formal banking index	0.42	0.31	0.53	0.000	1510	0.000
Economic status index	−0.04	−0.15	0.07	0.503	1510	0.418
Confidence index	0.01	−0.09	0.11	0.842	1510	0.711
Attitudes of social empowerment index	0.07	−0.04	0.18	0.242	1510	0.031
Perceived norms of social empowerment index	−0.01	− 0.13	0.10	0.865	1510	0.188
Relationship status (ended: y/n)	−0.01	−0.05	0.04	0.617	1625	NA
Independent decision-making index	0.11	0.00	0.21	0.028	1325	0.007
IPV index (0–11 count)	0.06	−0.09	0.21	0.453	1336	0.190
Checkup Measures						
STI test (y/n)	0.03	0.00	0.06	0.134	1129	NA
Family planning (y/n)	0.03	−0.02	0.07	0.134	1136	NA
Stress (0–4)	−0.04	−0.11	0.04	0.317	1170	NA
Depression (0–4)	−0.10	−0.18	− 0.02	0.012	1168	NA
Anxiety (0–4)	−0.04	−0.14	0.05	0.424	1167	NA
Systolic blood pressure	−0.71	−1.87	0.44	0.230	1144	NA
Diastolic blood pressure	0.45	−0.56	1.46	0.384	1144	NA
Injury (y/n)	0.01	−0.01	0.03	0.317	1170	NA
Violence (0–7 count)	−0.02	−0.14	0.10	0.741	1165	NA
Violence frequency (0–4)	−0.03	−0.27	0.21	0.803	1170	NA

We present the coefficient for the effect of the savings treatment on each dependent measure, the 95% confidence interval for the treatment coefficient based on robust standard errors, the *p* value for the treatment coefficient, and the number of observations for each dependent measure. For dependent measures that are standardized indices corresponding to a family of related survey measures, we analyze the index components using seemingly unrelated regression (SUR) and conduct a Wald test of the significance of the treatment coefficients within the system of equations; the *p* value for this joint test of significance is presented in the final column. We control for baseline assessment of dependent measures, socioeconomic status, life stage, and project site. Numeric ranges in parentheses refer to the response scale of dependent measures; y/n indicates a binary yes/no response scale

### Heterogeneous effects

**Fig. 2:**
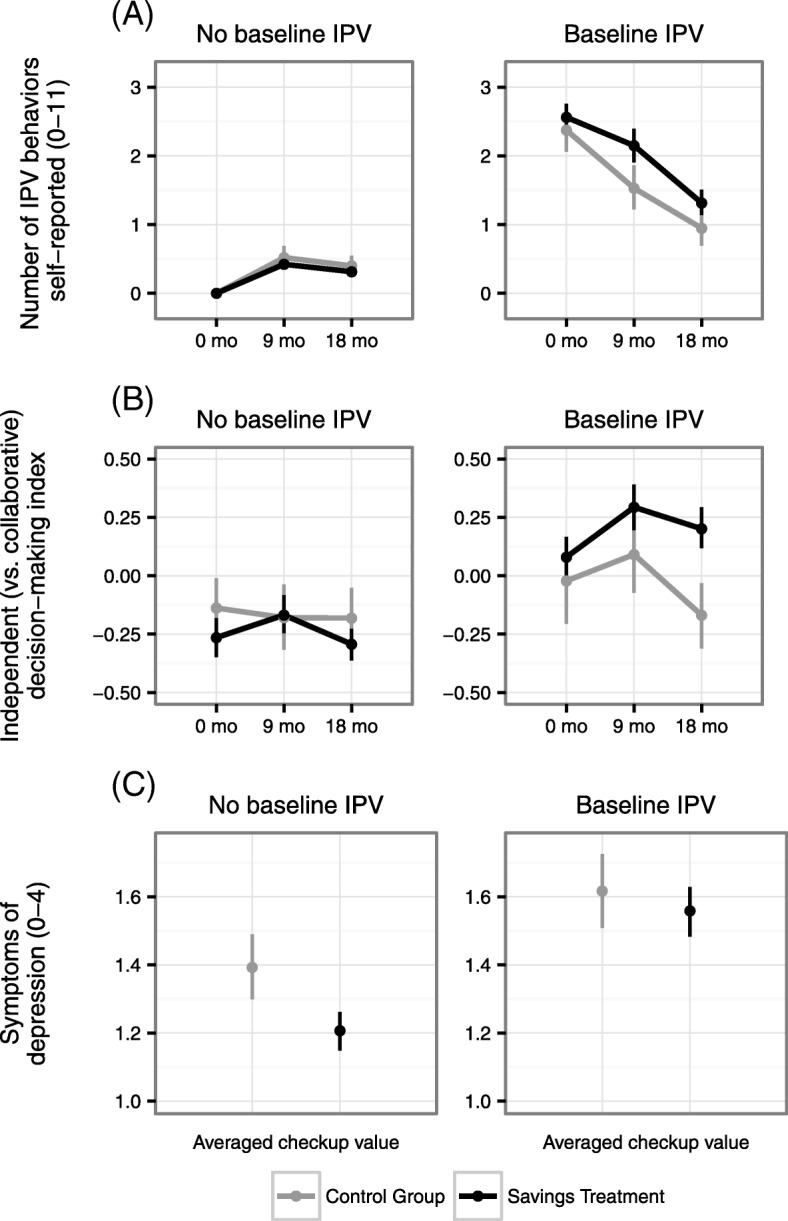
Heterogeneous treatment effects by baseline IPV. Treatment effects among participants who did versus did not self-report intimate partner violence (IPV) at baseline, for self-reported IPV victimization (Panel **a**; non-standardized total count of violent behaviors), independent (vs collaborative) decision-making (Panel **b**; standardized weighted index), and symptoms of depression (Panel **c**; non-standardized mean score). Panels **a** and **b** depict survey measures at 0, 9, and 18 months; Panel **c** depicts averaged post-treatment health service measurements (taken up to 3 times from 0 through 18 months). Point estimates with 95% confidence intervals are presented

The effects of the bundled savings treatment on several key outcomes depended on whether women’s partnerships were free of violence when they entered the trial. We found statistically significant and in some cases substantive heterogeneous treatment effects of the bundled savings treatment on perceived norms, decision-making patterns in relationships, self-reported IPV victimization, and health (see Fig. 2).

For women reporting no IPV at baseline, the bundled savings treatment did not affect decision-making with their partners or IPV victimization, although it caused stronger perceived norms of social empowerment (B = 0.14, 95% CI = − 0.02, 0.29), and lower symptoms of depression (B = − 0.16, 95% CI = − 0.27, − 0.05) and stress (B = − 0.10, 95% CI = − 0.20, 0.00) in the health services, relative to the control group.

However, for women reporting IPV victimization at baseline, the bundled treatment caused weaker perceived norms of social empowerment (B = − 0.21, 95% CI = − 0.37, − 0.04), higher levels of independent (vs collaborative) decision-making (B = 0.33, 95% CI = 0.15, 0.51), greater stability over time in their reported levels of IPV (B = 0.39, 95% CI = 0.07, 0.72), and greater use of family planning in health services (B = 0.09, 95% CI = 0.01, 0.16); it did not affect depression or stress. See Table 3 for all heterogeneous treatment effects by baseline IPV victimization.

**Table 3:** Heterogeneous treatment effects by baseline IPV

Dependent Measure	Simple Effect of Treatment (Participants Reporting No Baseline IPV)					Simple Effect of Treatment (Participants Reporting Baseline IPV)					Interaction Between IPV and Treatment				Interaction
	B	[95% CI	95% CI]	p	N	B	[95% CI	95% CI]	p	N	B	[95% CI	95% CI]	p	Joint p
Survey Measures															
Formal banking index	0.41	0.27	0.56	0.000	875	0.42	0.25	0.60	0.000	635	0.01	− 0.22	0.23	0.936	0.830
Economic status index	−0.08	− 0.22	0.06	0.254	875	0.03	−0.15	0.21	0.741	635	0.10	−0.12	0.33	0.363	0.123
Confidence index	0.07	−0.05	0.19	0.242	875	−0.05	−0.22	0.12	0.576	635	−0.12	−0.33	0.09	0.276	0.268
Attitudes of social empowerment index	0.02	−0.12	0.17	0.772	875	0.12	−0.05	0.29	0.184	635	0.12	− 0.10	0.34	0.276	0.325
Perceived norms of social empowerment index	0.14	−0.02	0.29	0.080	875	−0.21	−0.37	− 0.04	0.009	635	−0.33	− 0.55	−0.10	0.006	0.012
Relationship status (ended: y/n)	0.02	−0.04	0.07	0.503	927	−0.04	−0.12	0.04	0.317	698	−0.06	−0.15	0.04	0.230	NA
Independent decision-making index	−0.06	− 0.20	0.07	0.390	795	0.33	0.15	0.51	0.000	530	0.40	0.18	0.63	0.000	0.011
IPV index (0–11 count)	− 0.10	−0.26	0.06	0.211	799	0.39	0.07	0.72	0.022	537	0.49	0.13	0.85	0.007	0.041
Checkup Measures															
STI test (y/n)	0.04	−0.01	0.08	0.046	640	0.02	−0.03	0.07	0.503	489	− 0.01	− 0.07	0.06	0.741	NA
Family planning (y/n)	−0.02	− 0.08	0.04	0.503	641	0.09	0.01	0.16	0.024	495	0.11	0.02	0.20	0.028	NA
Stress (0–4)	−0.10	−0.20	0.00	0.046	664	0.04	−0.08	0.16	0.503	506	0.14	−0.01	0.28	0.080	NA
Depression (0–4)	−0.16	−0.27	− 0.05	0.001	662	−0.02	− 0.15	0.10	0.772	506	0.14	−0.03	0.30	0.080	NA
Anxiety (0–4)	−0.10	−0.23	0.03	0.153	662	0.02	−0.12	0.17	0.772	505	0.13	−0.06	0.32	0.194	NA
Systolic blood pressure	−0.48	−2.06	1.10	0.555	649	−0.85	−2.50	0.80	0.313	495	−0.41	−2.65	1.84	0.719	NA
Diastolic blood pressure	0.18	−1.19	1.56	0.795	649	0.79	−0.65	2.22	0.280	495	0.72	−1.25	2.69	0.472	NA
Injury (y/n)	0.00	−0.03	0.04	1.000	664	0.02	−0.02	0.06	0.317	506	0.01	−0.04	0.06	0.741	NA
Violence (0–7 count)	−0.01	−0.13	0.12	0.865	661	−0.06	−0.26	0.15	0.582	504	−0.07	−0.30	0.17	0.562	NA
Violence frequency (0–4)	−0.18	−0.43	0.08	0.168	664	0.12	−0.33	0.57	0.603	506	0.31	−0.18	0.81	0.215	NA

We present simple effects of treatment among participants who did and did not, respectively, report recent intimate partner violence (IPV) at baseline. We then present the interaction between treatment and baseline IPV, from a separate regression in which effects of treatment and baseline IPV were included. For each effect, we present the coefficient for each dependent measure, the 95% confidence interval for the coefficient based on robust standard errors, the *p* value for the coefficient, and the number of observations for each dependent measure. For dependent measures that are standardized indices corresponding to a family of related survey measures, we analyze the index components using seemingly unrelated regression (SUR) and conduct a Wald test of the significance of the interaction term coefficient within the system of equations; the *p* value for this joint test of significance is presented in the final column. We control for baseline assessment of dependent measures, socioeconomic status, life stage, and project site. Numeric ranges in parentheses refer to the response scale of dependent measure; y/n indicates a binary yes/no response scale

## Discussion

A bundled savings treatment produced a small increase in all women’s economic empowerment (in terms of formal financial participation) and decrease in symptoms of depression, but no overall change in their social empowerment (in terms of confidence, perceptions of her partnership or of norms regarding women in her community), or experience of IPV. One interpretation of this result is that the economic impact was too small to open up new possibilities in women’s lives. Another interpretation is that economic empowerment affected different types of women in different ways. Support for this latter interpretation comes from the finding that women who started the project in less violent relationships showed some evidence of social empowerment, though these effects were small and restricted to only some measures. By contrast, women who reported experiencing violence showed no evidence of increased social empowerment. These women reacted to the treatment by making more independent decisions within their relationships, but this did not facilitate their influence within their relationship or decrease levels of relationship violence over time, relative to the control group.

These heterogeneous findings align with other economic and social empowerment interventions for women that discovered heterogeneous or context-dependent effects [1–5]. For example, cash stipends improved long-term economic outcomes only for young women in settings where there are economic opportunities [1]. The conditional causal effects in our sample strongly suggest similarly heterogeneous effects of savings accounts on social outcomes such as decision-making and IPV.

Our finding of reduced symptoms of depression is consistent with evidence that increased financial security can improve psychological wellbeing [7]. Considering that the effect on symptoms of depression was largest among participants who did not report IPV at the start of the study, it is also possible that positive feelings were inspired by the increase in perceived norms of social empowerment, or by the experience of taking on a new endeavor with their partner.

The results of this study suggest that even economic interventions that improve the financial wellbeing of individual women will not always shift women’s social empowerment specifically within their families. To do this, an intervention may need to change not just the woman’s psychology––her patterns of thinking or feeling––but also her patterns of social and economic interaction. Interventions aimed at these broader effects might need to incorporate large cash influxes paired with accounts so that women control the money [7], peer groups who participate along with the woman [19], or a program that coaches a couple’s teamwork in a financial venture. Future studies could also assess the potential of economic interventions for primary prevention of IPV, by testing program effects on the trajectory of women’s new relationships.

The present study is one of the few economic interventions that has been conducted in poor urban communities with high levels of community violence and low trust in banks. These features may be important for understanding the study’s findings and the limits on their generalizability. Our findings suggest the need to continue developing and testing strategies for addressing IPV in such settings. In addition, the eligibility criteria used to select participants may limit the generalizability of the findings. Other limitations include the low uptake of the savings treatment and the irregularity of attendance at the health checkups offered within the study.

## Conclusions

Economic interventions have recently caught the attention of public health researchers interested in social empowerment and intimate partner violence, thanks to the success of trials that have demonstrated widespread effects of cash transfers on social and health outcomes [2, 5]. Our research sounds a cautionary note on two grounds. One, it is consistent with growing evidence that economic interventions may need to lead to immediate and substantial economic benefit to have broader impacts. Two, it suggests that economic interventions alone cannot address the multitude of relational and contextual factors that shape social empowerment and violence [2, 5].

Even though our research does not support the grandest claims for economic interventions, it does support their limited utility. Economic empowerment is an important part of the bundle that can increase women’s social empowerment and reduce IPV. For example, our findings and others indicate that in the context of supportive relationships and other economic opportunities, even small and short-term economic infusions have positive social and health effects [1–5]. It remains for future research to explore how to bundle economic empowerment with other elements to improve women’s lives.

## Additional file

Additional file 1: Contains additional information regarding method, treatment effects, retention and take-up, dependent measures, and other study details (PDF 1414 kb)

## Abbreviations

CI: Confidence Interval
CONSORT: Consolidated Standards of Reporting Trials
IPPF: International Planned Parenthood Federation
IPV: Intimate Partner Violence
IRB: Institutional Review Board
SES: Socioeconomic Status
SMS: Short Message Service
STI: Sexually Transmitted Infection
SUR: Seemingly Unrelated Regression
USD: U.S. Dollars
WHR: Western Hemisphere Region

## Footnotes

1 The study’s eligibility criteria defined “poor” based on being low-income and living in a neighborhood classified by the government as low in social class. See Supplementary Material for further information regarding eligibility criteria.

2 Some follow-up survey interviews took place outside the home (e.g., at the clinic, at a church) due to changing safety conditions in the communities, and participants were offered the option of scheduling the 18-month survey outside the home for privacy. A total of 151 of the 9-month surveys and 227 of the 18-month surveys were implemented outside participant homes.
