# Supplementary material for: The effect of a savings intervention on women’s intimate partner violence victimization: heterogeneous findings from a randomized controlled trial in Colombia
Source: BMC Womens Health. 2019 Jan 25;19:17. doi: 10.1186/s12905-019-0717-2 (PMC6347741; doi:10.1186/s12905-019-0717-2)
Supplement: Supplementary file 1 — Contains additional information regarding method, treatment effects, retention and take-up, dependent measures, and other study details (PDF 1414 kb) [file 12905_2019_717_MOESM1_ESM.pdf]

Additional file 1

A. **Method**
B. **Treatment Effects**
C. **Retention and Take-up**
D. **Dependent Measures**
E. **Additional Study Details**
F. **References**

A. Method

Pre-registration

Registered retrospectively, prior to realization of outcomes, 5/29/14: Evidence in Governance and Politics, ID #20140529AA

Replication Dataset, Codebook, and Analysis Code

Open Science Framework
https://osf.io/qwnp3/?view_only=bad5ca8fafbc4d2d9945872e7c7f1c49

Study Procedure: Additional Details

Power Analysis. Power analysis was conducted based on savings product takeup rates from previous research (Ashraf et al., 2010), the effect size in research analyzing the addition of a group-based education and discussion component to a financial intervention (Kim et al., 2009), and analyses planned for comparing the three treatment groups.

Recruitment, Randomization, and Blinding. 1800 female participants were recruited across four low-income neighborhoods in Cali, Palmira, and Buenaventura, Colombia. Survey enumerators from a Colombian survey firm enrolled participants in the study. Each site contained a branch of the project's partnering bank and health clinic (Profamilia, the Colombian affiliate of the International Planned Parenthood Federation, Western Hemisphere Region).

The oral consent script was approved by Princeton Institutional Review Board, Protocol #6116.

Participants were randomly assigned at the individual level to one of three treatment groups. One group received the savings treatment combined with personal messaging (N = 685), one received the savings treatment combined with normative messaging (N = 679), and a control group received neither the savings treatment nor a messaging treatment (N = 436). In the present study, we collapse the two messaging treatment arms to compare the 1364 participants assigned to the savings treatment with the 436 participants assigned to the control group.

The random allocation sequence was generated by the research team using random number generation in Microsoft Excel. The researchers provided the sequence to the survey firm, and supervisory staff at the firm wrote a number on the back of each ordered survey indicating the assigned treatment group, so that the survey enumerator and participant would be blinded to the assigned group prior to reaching the end of the baseline survey interview. The survey enumerator was required to implement the surveys in the order in which they were provided, and not permitted to view the assigned group ahead of time. The research team and survey firm supervisory staff emphasized the research importance of these requirements during training. Additionally, the survey firm required that at least 5% of surveys be monitored by a supervisor during at least 75% of the interview (in addition to other quality control measures to verify responses).

Staff at Profamilia and at the bank were not informed of the details of the study design, but were inherently aware that participants they interacted with were receiving the services that the staff provided. It is possible that some participants referenced other aspects of the study to the staff. Participants were aware of the services that they themselves were receiving, and were aware that other participants might be receiving different types of services, but were unaware of the details of the different services.

Qualitative Research. Qualitative research took place at various points before, during, and after the project, to supplement the quantitative measures and aid in interpreting them. The qualitative components were designed to deepen the research team's understanding of the effects of the experimental treatments and augment the quantitative data.

Savings Intervention: Additional Details

The visual aid introducing participants to the savings account explained that the 18-month project sought to motivate women to save, and that the participant would be able to open a savings account at the partnering bank. It reminded participants that saving in an account can help in working toward goals and moving a family forward. It explained that the account is free, belongs to the participant, and allows deposits and withdrawals at any time. It then stated that Proyecto Crecer offers certain incentives to motivate the participant to save in the account, and that if one saved at least 20,000 pesos (~$10 USD) during 3 months, she would receive 20,000 pesos as a prize from the project for saving well. The enumerator asked if the participant wanted to open the account. Colombian regulatory and related logistical factors prevented opening the accounts in the home during the enumerator's visit.

B. Treatment Effects

Analytic Strategy

Survey Measures. We analyzed dependent measures as assessed in the endline (18-month) survey.

Checkup Measures. We computed each participant's average response value for each outcome measure, across the number of checkups that the participant attended. Among the 1170 participants who attended at least one checkup, 331 (28%) attended one checkup, 294 (25%) attended two, 542 (46%) attended three, and 3 (.3%) attended four. Five-hundred ninety-four participants (51%) attended a checkup during the first half of the project (on or before March 3, 2014); the other 576 who attended at least one checkup did not attend any during the first half of the project.

Effect Estimation

We conducted intention-to-treat analyses, analyzing all responses recorded based on savings treatment assignment, regardless of whether a participant took up the savings account offer. We used linear regression in the results presented; patterns were consistent when using logistic regression for two dependent measures that are binary.

Average Treatment Effects (Table 2 in main text)

We first tested the effect of the savings treatment on each dependent measure. The model is as follows:

$$Y_{End_i} = B_1 X_i + B_2 Y_{Base_i} + B_3 S_{1i} + B_4 S_{2i} + a_j + e_i$$

The dependent variable for the individual i is Y_{End_i} and the baseline measure of this variable (or a close proxy, where not measured at baseline) is Y_{Base_i}. The savings treatment is X_i and we present the effect of treatment (B_1) for each dependent measure in Table 2.

Baseline covariates are a standardized index of socioeconomic status (S_{1i}, based on neighborhood modal social class, income, subjective SES, work status, and education level) and a standardized index of life stage (S_{2i}, based on age, having children, and being married or in a civil union). For socioeconomic dependent variables, we excluded S_{1i} from the model, given its redundancy with Y_{Base_i}. For the checkup measures, we additionally controlled for the number of checkups that a participant attended and whether or not the participant attended any checkups during the first half of the project.

Fixed effects of project site j are a_j (with Buenaventura as the reference group) and the error term is e_i. We estimated robust standard errors.

Heterogeneous Effects (Table 3 in main text)

We then tested whether the effects of financial treatment differed depending on baseline self-reports of IPV. Specifically, we interacted condition assignment with a dummy variable indicating whether a participant self-reported experiencing any of the 11 listed violent behaviors from her partner

in the baseline survey. At baseline, 43% of participants (768 out of 1800) reported experiencing any of the 11 listed violent behaviors. The model is as follows:

$$Y_{End_i} = B_1 X_i + B_2 V_{Base_i} + B_3 X_i * V_{Base_i} + B_4 Y_{Base_i} + B_5 S_{1i} + B_6 S_{2i} + a_j + e_i$$

A dummy variable coding whether the participant reported one or more of the 11 IPV behaviors at baseline is V_{Base_i}. For the dependent measure of IPV, we excluded Y_{Base_i} from the model, given its redundancy with V_{Base_i} for these variables. The interaction term between baseline IPV and financial treatment is $X_i * V_{Base_i}$ and we present the term's coefficient (B_3) in Table 3. We estimated robust standard errors.

Baseline self-reports of IPV as a moderator hold up to controlling for other hypothesized potential interactions between key baseline indices (confidence, socioeconomic status, and trust in institutions) and treatment.

Relationship-Relevant Dependent Measures

Dependent measures related to independent decision-making and IPV were based on questions about the participant's partner or relationship dynamics. In the 18-month survey, to minimize missing data, all participants were asked to respond to these questions, regardless of current relationship status. For these questions, any participant who was not currently in a relationship was asked once early in the survey if she would like to respond about her last partner just before breaking up, her last partner after breaking up, or a new person who is not her partner yet.

We excluded only responses about the last partner before breaking up, given that these responses reflect past interactions whereas the other responses reflect current interactions (with the last partner or a new person). Including responses about a participant's last partner just before breaking up did not substantially change the overall pattern of results; the (non-robust) average treatment effect on independent decision-making was not robust to including these responses.

Among the 14% (218 / 1510) of participants in the 18-month survey who did not identify as being in a relationship, 80% chose to respond about their last partner just before breaking up, 18% chose to respond about their last partner after breaking up, and 2% chose to respond about a new person who was not their partner yet.

Interim Analyses

We analyzed the data from each survey wave after the wave was completed, including the baseline survey and 9-month survey. Analysis of the baseline survey informed our understanding of the sample, which helped in tailoring messaging about the intervention to this population. Analysis of the 9-month survey provided preliminary findings to ensure that participant safety was being protected and to assess whether exploratory measures should be added to the 18-month survey to better understand the pattern of results. Pre-registration took place between the baseline and 9-month surveys.

C. Retention and Take-up

Retention

Table S1. Retention by data collection wave and condition

Data Collection Wave	Dates	Condition	Number of Participants Observed	Proportion of Baseline Sample Observed
Baseline Survey	June 2, 2013 –	Control	436	–
(0 Months)	October 1, 2013	Treatment	1364	–
		TOTAL	1800	–
Midline Survey	March 11, 2014 –	Control	405 / 436	0.929
(9 Months)	June 25, 2014	Treatment	1207 / 1364	0.885
		TOTAL	1612 / 1800	0.896
Endline Survey	March 2, 2015 –	Control	383 / 436	0.878
(18 Months)	June 19, 2015	Treatment	1127 / 1364	0.826
		TOTAL	1510 / 1800	0.839
Health Checkup	June 2, 2013 –	Control	341 / 436	0.782
	May 31, 2015	Treatment	829 / 1364	0.608
		TOTAL	1170 / 1800	0.650

Note. For health checkups, we include the number of participants who attended at least one of the three checkups offered.

Take-up

Among the 1364 participants assigned to the savings treatment, 690 (49.3% of 1364) opened the account at some point during the project and 455 (33.4% of 1364) made a deposit in their account at some point during the project.

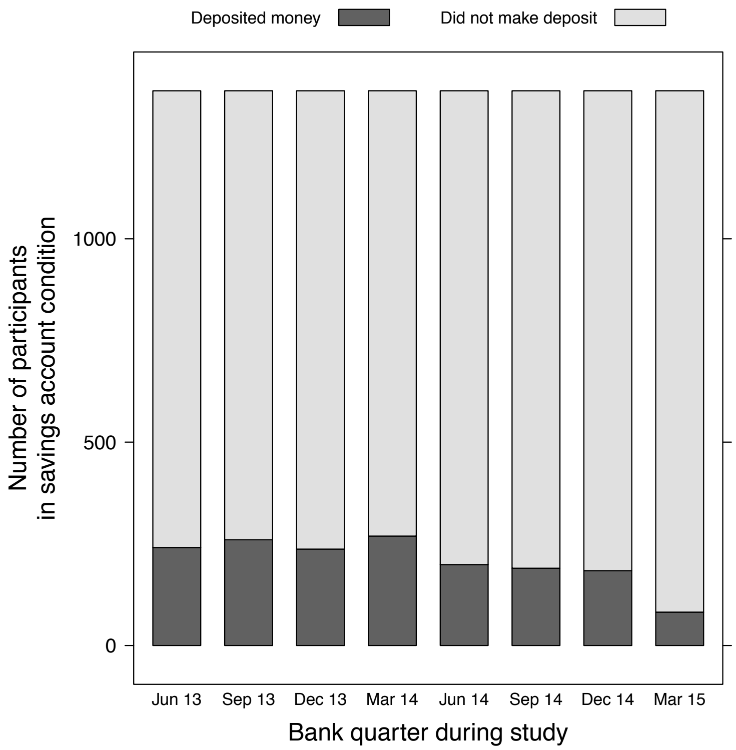

Figure S1. Account deposits across quarterly periods of project

D. Dependent Measures

We measured different aspects of participants' financial engagement, empowerment, and experience of IPV. Most items were answered on a four-point scale, presented as agree-or-disagree questions with a single follow-up question asking about the strength of the participant's agreement or disagreement. For sets of items planned to serve as scales, we calculated mean scores and report Cronbach's α as a measure of the scale's reliability. We generated standardized index variables for each family of outcomes based on factor analysis. We selected the first factor in the factor solution. The letter (R) indicates when an item or score was reverse-scored in the corresponding index.

In the baseline survey, additional information was collected about the participant's household, family structure, relationship history, income, personal goals, education, other demographic characteristics, and potential psychological moderators such as identification with women in the community as a group.

Within the main text, all effects presented correspond to versions of these variables that have been standardized based on the control group of the respective wave (where dependent measures were not already standardized weighted indices).

The outcome areas included in our pre-registration remained the same after the trial commenced. Some modifications to measures were made based on feedback from the survey enumerators regarding interpretability of items to participants. Some items were also added based on feedback from the survey enumerators, feedback from staff at the partnering bank and health clinic, and qualitative findings, to better capture participants' experience with the project.

Table S2. Survey Measures

**Dependent Measure**	**Items**
Formal banking standardized weighted index	*Trust in banks*. Participants' trust in banks was measured using the following two items based on other scales of trust in institutions (e.g., Devos, Spini, & Schwartz, 2002): "Do you trust in banks?" and "Do you feel comfortable interacting with people who work in banks?" (1=not at all, 4=a lot; Cronbach's α = .80).<br><br>*Negative perceptions of banks (R).* Participants' negative perceptions of banks were measured using the following two items developed based on preliminary qualitative research: "Banks are just trying to take your money" and "An account means I cannot get benefits from the government or I may be taxed" (1=not at all in agreement, 4=very much in agreement; Cronbach's α = .74).<br><br>*Difficulty going to bank (R).* Participants' perceived difficulty of going to the bank was measured with the following question: "How difficult does it feel to go to a bank?" (1=not at all difficult, 5=haven't tried).<br><br>*Formal saving.* Participants' self-reported formal saving behavior in the last six months was measured for each of three purposes ("small, everyday expenses," "future expenses such as education, a wedding, or a big purchase," and "emergencies or a time when you expect to have less income"). Participants were asked whether they had saved money at a bank for each of the three saving purposes (Cronbach's α = .78).

Economic status standardized weighted index	*Income.* Participants indicated the average monthly income that they receive in Colombian pesos, adding up all sources including a family member, informal work, formal work, an informal business, a formal business, and other sources. This amount was log-transformed (after adding a value of "1" to each response to be able to retain responses of "0" in the transformation).<br><br>*Subjective SES.* As a measure of subjective socioeconomic status (SES), participants indicated where they would place themselves on a ladder representing where people stand in Colombia (1=people "who are the worst off—who have the least money, least education, and the least respected jobs or no job," 10=people "who are the best off—those who have the most money, the most education, and the most respected jobs"; adapted from Adler, Epel, Castellazzo, & Ickovics, 2000).<br><br>*Work status.* Participants indicated their current work status. We coded whether they were working (stable or unstable work) as opposed to looking for work, unemployed and not looking for work, housewife and not looking for paid work, disabled (unable to work), or another work status.
Confidence standardized weighted index	*Self-efficacy.* Self-efficacy (Bandura, 1986) was measured as it related to confidence in being able to access services and resources under challenging life circumstances, through three items (e.g., "In the event of a crisis in the home, would you feel capable of raising enough money to feed your family for 4 weeks, if it were your responsibility?" and "If your partner were mistreating you physically or emotionally, would you feel capable of finding where to go if it were necessary?"; 1=not at all sure, 4=very sure; Cronbach's α = .46; adapted from Kim et al., 2007).<br><br>*Self-esteem.* Self-esteem was measured using four items from Heatherton and Polivy's (1991) state self-esteem scale (e.g., "Do you feel confident about your abilities?" and "Are you pleased with your appearance right now?"; 1=not at all, 4=a lot; Cronbach's α = .62).<br><br>*Future time perspective.* Future time perspective (FTP), a dimension that is positively related to constructs such as optimism and life satisfaction (Brothers, Chui, & Diehl, 2014), was measured using six items from Carstensen and Lang's (1996) scale (e.g., "Do you believe that the future is full of possibilities?" and "Do you expect that you will set many new goals in the future?"; 1=not at all, 4=a lot; Cronbach's α = .83).
Attitudes of social empowerment standardized weighted index	We measured personal attitudes and perceived norms regarding support for following a partner's wishes and regarding justification of IPV. We created separate indices for attitudes and norms.<br><br>While the index components correspond to "low" attitudes of social empowerment (for example, higher values for tolerance of IPV correspond to greater tolerance of IPV), the index was reverse-scored (multiplied by negative one) to correspond to "high" attitudes of social empowerment (higher index values correspond to lower support for following of partner's wishes and lower justification and tolerance of IPV).<br><br>*Following partner's wishes.* Participants responded to sets of questions about behavior and attitudes regarding following the wishes of a partner. Within each question set, they were asked about the following 4 behaviors: telling a partner about money one has saved, following a partner's decisions about family planning, telling a partner who one spends time with, and following the wishes of a partner when making decisions about education, life conditions, and work.<br>• *Following partner's wishes: behavior.* Participants indicated how frequently they engage in each of the 4 behaviors toward their partner (4 items; e.g., "With what frequency do you tell your husband or partner about money you have saved?"; 1=almost never, 4=almost always; Cronbach's α = .80).<br>• *Following partner's wishes: attitudes.* Participants indicated whether they think women should engage in each of the 4 behaviors toward their partner (4 items; e.g., "Do you think women should tell their husband or partner about money they have saved?"; 1=not at all, 4=a lot; Cronbach's α = .70).<br><br>*Justification of IPV: attitudes.* Participants responded to four items about whether a man has a good reason for hitting his partner based on 4 different behaviors in which she might engage (e.g., "Does a man have a good reason for hitting his partner if she neglects taking care of the children?"; 1=not at

	all, 4=a lot). The 4 behaviors were neglecting taking care of the children, not having sex with him, not preparing the food, and going out without telling him (Cronbach's α = .82).<br><br>*Tolerance of IPV: attitudes.* Participants responded to the following single item: "Do you think women should tolerate violence from their partner to keep the family together?" (1=not at all, 4=a lot).
Perceived norms of social empowerment standardized weighted index	As was the case for attitudes of social empowerment, while the index components correspond to "low" perceived norms of social empowerment (for example, higher values for tolerance of IPV correspond to greater perceived tolerance of IPV), the index was reverse-scored (multiplied by negative one) to correspond to "high" perceived norms of social empowerment (higher index values correspond to lower perceived norms of following of partner's wishes and lower perceived justification and tolerance of IPV).<br><br>*Following partner's wishes.* Participants responded to sets of questions about perceived descriptive and prescriptive norms regarding following the wishes of a partner. Within each question set, they were asked about the same 4 behaviors as in the corresponding question sets in the personal attitudes index (above).<br>• *Following partner's wishes: perceived descriptive norms.* Participants indicated how frequently women in their community engage in each of the 4 behaviors toward their partner (4 items; e.g., "With what frequency do women in your community tell their husband or partner about money they have saved?"; 1=almost never, 4=almost always; Cronbach's α = .73).<br>• *Following partner's wishes: perceived prescriptive norms.* Participants indicated whether women in their community think women should engage in each of the 4 behaviors toward their partner (4 items; e.g., "Do women in your community think women should tell their husband or partner about money they have saved?"; 1=not at all, 4=a lot; Cronbach's α = .76).<br><br>*Perceived change in norms among women (R).* After responding to the questions about attitudes and norms surrounding following a partner's wishes, participants were asked about whether they think women's attitudes and men's attitudes on these topics are changing. We used the following two items to measure perceived change in women's support for having control over their finances and lives: "When you think about women having their own money and deciding how to spend it the way they want, are women's attitudes becoming more supportive, less supportive, or are they not changing?" and "When you think about women being able to make their own decisions about issues like family planning, free time, their education, and their work, are women's attitudes becoming more supportive, less supportive, or are they not changing?" (1=support much less, 3=no change, 5=support much more; Cronbach's α = .58).<br><br>*Justification of IPV: perceived prescriptive norms.* Participants responded to 4 items about whether women in the community think a man has a good reason for hitting his partner based on 4 different behaviors in which she might engage (e.g., "Do women in your community think a man has good reason for hitting his partner if she neglects taking care of the children?"; 1=not at all, 4=a lot; Cronbach's α = .93). The 4 behaviors were the same as those for attitudes justifying IPV.<br><br>*Tolerance of IPV: perceived prescriptive norms.* Participants responded to the following single item: "Do women in your community think women should tolerate violence from their partner to keep the family together?" (1=not at all, 4=a lot).
Relationship status (ended: yes/no)	In the 9-month survey, participants were asked whether they were in the same relationship now as when they started the project. In the 18-month survey, participants were reminded of the month and year in which they were last surveyed, and asked if they remembered whether they had been in a relationship at that time. They then indicated whether they were in the same relationship now and, if the original relationship had ended, whether they were seeing a new partner now.<br><br>We analyzed relationship status at 18 months as well as reports that a relationship had ended in either the 9-month or 18-month survey wave, among participants who responded to one or both follow-up survey waves. Participants were asked about their relationship status at 9 months both during the 9-month survey and retrospectively during the 18-month survey.

Independent (vs collaborative) decision-making standardized weighted index	*Independent versus joint decision-making and initiation of discussions.* Participants responded to questions about how decisions are made in their household (adapted from Ashraf et al., 2010; Pitt, Khandker, & Cartwright, 2006). For five topics, participants were asked who makes most of the decisions (themselves, their partner, or both). The five topics were what to buy at the market, purchase of expensive items, recreational use of money, schooling of children, and visits to parents or other family members.<br>• We calculated the proportion of applicable decisions made independently (Cronbach's α = .80), as opposed to jointly or by the partner.<br>• Participants were also asked whether or not they themselves initiate discussions about each topic; we calculated the proportion of applicable decisions for which participants initiate discussions (Cronbach's α = .75).<br><br>*Independent decision-making about gift certificate.* At the end of the survey, a novel financial resource was introduced when participants were presented with a gift certificate as compensation for their time responding.<br>• They were asked whether or not they would tell their partner about the gift certificate (not telling him was coded as greater independent decision-making).<br>• They were also asked who would decide how to use the gift certificate (themselves, their partner, or both); we coded whether or not participants would decide independently. Less than 1% of participants indicated that their partner alone would decide and this response loaded onto a different factor than independent and joint decision-making.<br><br>*Partner's knowledge of money (R).* Participants indicated whether or not their partner "usually knows how much money [they] have, either at home or in [their] savings" (the partner not knowing how much money was coded as greater independent decision-making).<br><br>*Talking to partner about project (R).* Participants were asked if they have talked to different people on a list (e.g., partner, children, friends) about Proyecto Crecer. We coded whether or not they self-reported talking to their partner about it (not talking to him was coded as greater independent decision-making).
IPV victimization index (total behavior count)	For IPV victimization, we use an unweighted index for greater interpretability of effects. Specifically, the index is a count of the number of IPV behaviors in which the respondent's partner engaged. While this index is simplistic, the pattern is consistent when using a weighted index and is consistent across the different subtypes of IPV and a scale of relationship satisfaction.<br><br>Questions about intimate partner violence and relationship satisfaction were presented to the participant using a written card to allow for nonverbal responses.<br><br>*Intimate partner violence.* Participants were asked whether or not, in the past six months, their partner had engaged in each of 11 different violent and controlling behaviors (adapted from other studies investigating IPV internationally, e.g., Kim et al., 2007; Cronbach's α = .80). These behaviors were related to the following forms of violence:<br>• Financial violence (2 items; e.g., "taken your earnings or savings against your will"; Cronbach's α = .26).<br>• Emotional violence (5 items; e.g., "insulted you or made you feel bad about yourself"; Cronbach's α = .69).<br>• Physical violence (2 items; e.g., "thrown something at you, slapped you, pushed you, or crushed you"; Cronbach's α = .69).<br>• Sexual violence (2 items; e.g., "physically forced you to have sexual relations against your will"; Cronbach's α = .77).<br>For each of these 4 types of violence, we generated a count of the number of behaviors reported. We then calculated the total number of IPV behaviors reported across all 11 items.
Additional measures in Table 1	*Number of recent stressful events*. Participants were asked if any of the following seven events had happened to them in the past year: change of work; death of family member or friend; abortion or infant death; surgery, sickness, or injury; someone arriving in or leaving the home; problems with the family of one's partner; or another stressful event.

(baseline demographics)	*Frequency of talking to neighbors*. Participants were asked how often they talk to their neighbors, from 1 (never) to 6 (several times per day).<br><br>*Neighborhood SES level*. The participant's neighborhood's level on Colombia's 6-class system (DANE, 2013) was documented.<br><br>*Current formal financial services*. Participants were asked if they have ever had, and if they still have, any of the following financial services: formal loan, salary disbursement account, credit card at bank, credit card at retail store.<br><br>*Putting money side past 6 months*. Participants were asked whether, in the past 6 months, they have set aside *(guardar)* any money.<br><br>*Saving for purpose past 6 months*. Participants were asked whether, in the past 6 months, they have saved *(ahorrar)* any money for a specific goal.<br><br>*Identification with Colombians*. Participants responded to 3 items regarding feeling connected with, identifying with, and feeling solidarity with Colombians (e.g., "Do you feel connected with Colombians?". They responded on a scale from 1 (not at all) to 4 (a lot).<br><br>*Identification with women in community*. Participants responded to 3 items regarding feeling connected with, identifying with, and feeling solidarity with women in their community (e.g., "Do you feel connected with women in your community?". They responded on a scale from 1 (not at all) to 4 (a lot).

Table S3. Health Checkup Measures

**Dependent Measure**	**Items**
STI test	Participants were offered a test for sexually transmitted infections (STIs). The providers recorded whether the participant accepted or declined this service.
Family planning	Participants were offered a family planning consultation to discuss birth control methods. The providers recorded whether the participant accepted or declined this service.
Stress	Symptoms of stress were assessed by providers using the four-item version of Perceived Stress Scale (e.g., "In the last month, how often have you felt difficulties were piling up so high that you could not overcome them?"; 0=never, 4=very often; Cronbach's α = .70; Cohen, Kamarck, & Mermelstein, 1983; Cohen & Williamson, 1988).
Depression	Symptoms of depression were assessed by providers using the four Spanish items with the highest loading on each of four factors of the CES-D (e.g., "In the last week, how often have you felt that you enjoyed life?"; 0=never, 4=very often; Cronbach's α = .72; Losada et al., 2012; Radloff, 1977).
Anxiety	Symptoms of anxiety were assessed by providers using the four Spanish items with the highest loading on the Beck Anxiety Inventory (e.g., "In the last week, how often have you been affected by feeling shaky?"; 0=never, 4=very often; Cronbach's α = .76; Beck, Epstein, Brown, & Steer, 1988; Magán, Sanz, & García-Vera, 2008).
Systolic blood pressure	The providers recorded participants' blood pressure.

Diastolic blood pressure	The providers recorded participants' blood pressure.
Physical injuries	The clinicians recorded signs of physical injury identified on each part of the body (e.g., bruises, cuts), as in clinical IPV screening protocols (Basile, Hertz, & Back, 2007). We coded whether any injuries other than scars were recorded at any of a participant's checkups, given that scars do not necessarily indicate recent violence and the health clinic reported that many participants had scars from Cesarean sections.<br><br>Given the low rate of visible injuries documented during checkups, we computed whether any injuries were documented for a participant at any of her checkups, rather than computing the average value across checkups. An injury other than a scar was documented for only 4% (47) out of 1170 participants who attended a checkup.
Violence victimization self-reports	The clinicians also asked participants a series of self-report questions about experiences of emotional, physical, and sexual violence from their partner or "another person who is important in [their] life (father, children, siblings, etc.)." We counted the number of items (0-7) for which the participant indicated that violence had taken place or that she was at risk of violence (e.g., "At any time has your partner caused you physical harm, for example by kicking, punching, pushing, or hitting you?", "Are you afraid of your partner or someone else causing you harm?").
Violence victimization frequency	For each of 5 violence victimization items, the clinicians also asked how frequently the emotional, physical, and sexual violence took place ("How often has this happened?"; 0=never, 4=very often). We computed the mean score for these 5 items.

E. Additional Study Details

Table S4. Project eligibility requirements

Requirement type	Description
Neighborhood's modal social class (*estrato*)	1, 2, or 3, the lowest levels on Colombia's 6-class system (DANE, 2013)
Gender	Female
Age	18-55
Savings experience	Has not used in the last 12 months any service, product, or program to save with a financial institution, such as a bank or cooperative E.g., savings account, time-deposit account, ROSCA (*cadena*), savings cooperative, Fonda Nacional del Ahorro. Salary disbursement accounts, insurance, loans, credit cards, and other financial products and services were acceptable.
Experience with banking partner	Has not used in the last 12 months any service or product from the partnering bank (e.g., loan or account)
Similar savings program	Is not enrolled as a beneficiary of the program *Mujeres Ahorradoras en Acción* (a program by the Departamento para la Prosperidad Social that offers savings accounts with special conditions to women)
Relationship status	Currently has a partner who is male (cohabiting not required)
Income upper limit	Does not generally receive a monthly average of more than 2 million Colombian pesos (~$1000 USD) as money that she manages
Cell phone	Has her own cell phone that receives text messages
Interest in opening a savings account	Responds "yes" to the following question: "Consider for a moment your personal goals in the next years and what you would like to achieve. If you were presented with a free savings account to help you achieve your goals and this account were part of a project that would give you an incentive to encourage you to save, would you go to the bank to open the account?"

Note. Participants recruited during the first days of recruitment, June 2-7, 2013, were also required not to have used microcredit within the last 12 months. This eligibility requirement was lifted given the low rate of prospective participants who met it.

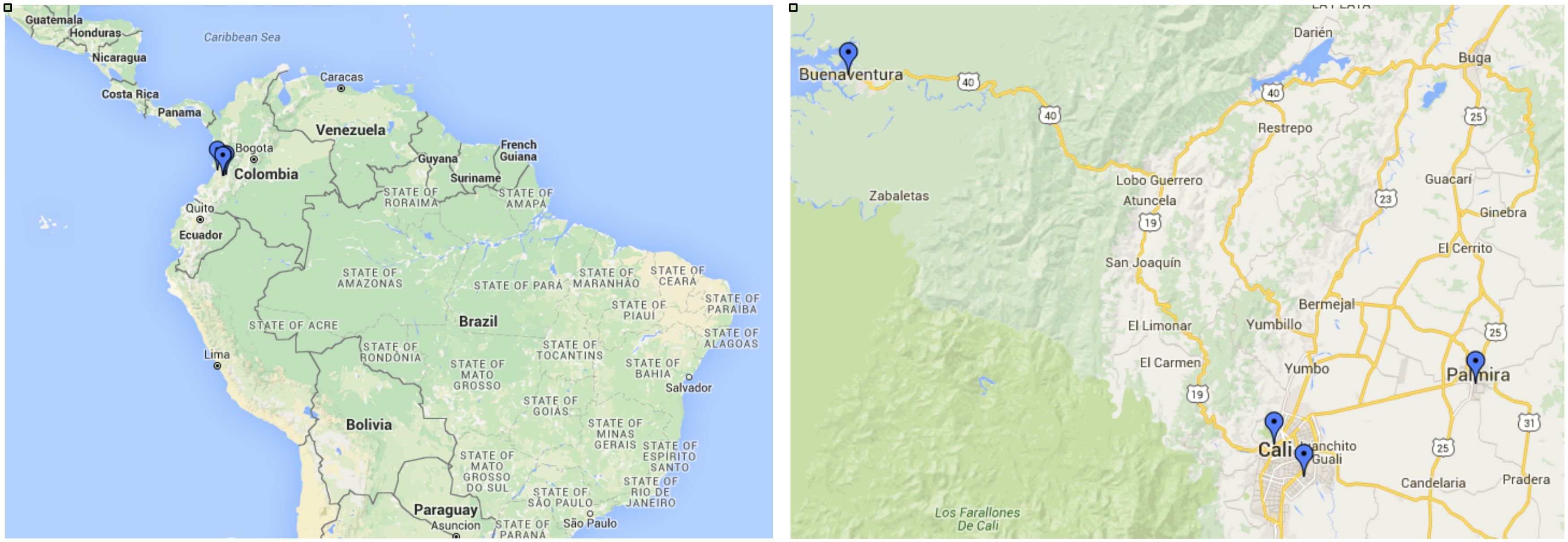

Figure S2. Map of four project sites in Valle del Cauca region of Colombia

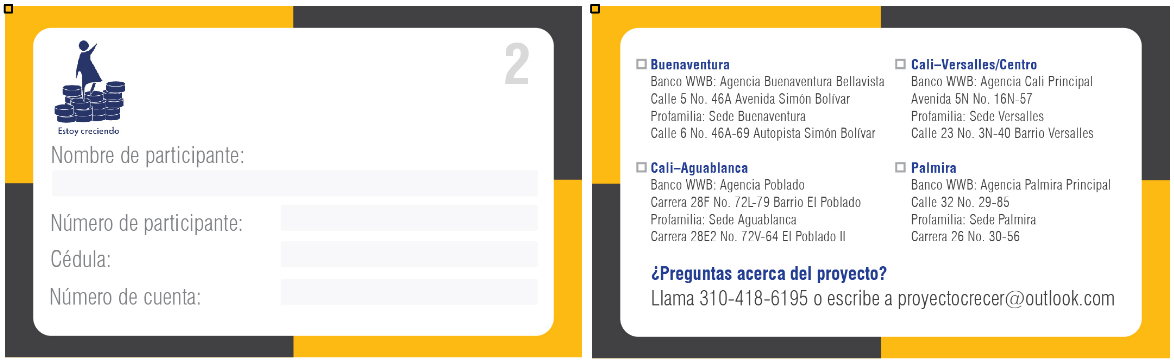

Figure S3. Participant ID card example (logo varied by treatment assignment)

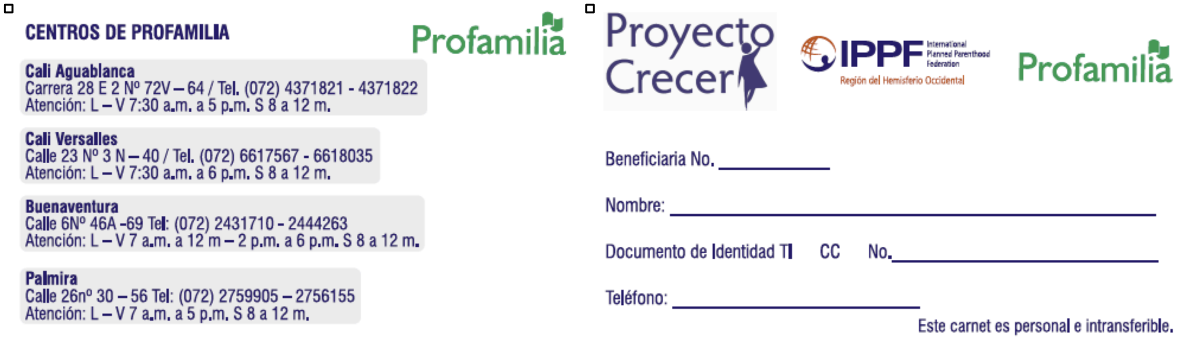

Figure S4. Profamilia health checkup voucher card

Table S5. Savings account incentives

Incentive	Launch date	Description
Seed money	June 2, 2013	Accounts were seeded with 10,000 pesos upon opening.
Quarterly deposit matching	June 2, 2013	Quarterly matching rate of 1/3 for savings account deposits, with matched amounts permitted to range from 20,000 pesos to 50,000 pesos per 3-month period (i.e., months 1 through 3, 4 through 6, 7 through 9, etc.). A deposit of at least 10,000 pesos needed to be made each month out of the 3 to qualify for that quarter's matching, and the participant did not qualify for a quarter's matching if she withdrew more than her account balance was at the beginning of that quarter.
Cell phone lottery	July 3, 2013	Participants were sent 1,000 pesos of cell phone minutes on their cell phone, and asked to reply "yes" to a code to participate in a lottery to win 10,000 pesos of cell phone minutes.
Gift	July 15, 2013	Participants received a small floral coin purse at the partnering bank upon opening the savings account.
New Year's lottery	December 23, 2013	Participants earned one lottery entry ticket for each 5,000 pesos deposited, up to 10 tickets per deposit. By writing their goals for the new year on the tickets, they could enter the tickets into the lottery. 1 prize of 500,000 pesos and 2 prizes of 250,000 pesos were awarded; participants were aware of the number and amount of the prizes.
Goal-planning lottery	March 11, 2014	Participants were presented with a goal-planning worksheet at the end of their midline survey, which they completed with the survey enumerator. Upon turning it in at the bank and having an open account, participants could be entered into a lottery for a prize of 5 million pesos. 7 prizes of this amount were awarded; participants were unaware of the number of prizes of this amount being awarded.
Savings tree scratch-and-win	October 1, 2014	Participants who made 3 deposits of at least 3,000 pesos each were given a scratch-and-win card to discover which prize they had earned (e.g., set of glasses, rice cooker, fan, toaster, TV, laptop). Upon making each deposit, the participant was given 1 of 3 paper puzzle pieces to add to a personal "savings tree" paper image.

Table S6. Example savings SMS reminders

Original Spanish Version	English Translation
Mujeres como tú saben que ahorrar con el Proyecto Crecer es un buen negocio para ellas, porque la plata que ponen en su cuenta del Banco WWB, produce más plata	*Women like you know that saving with Proyecto Crecer is a good business for them, because the money that they put in their Banco WWB account produces more money.*
Quien no pone un huevo, no saca un pollo. Las mujeres que le ponen platica a su cuenta del Proyecto Crecer en el Banco WWB, con constancia la aumentan.	*If you don't place an egg, you don't get a chicken. Women who put a little money in their Proyecto Crecer account at Banco WWB, they steadily increase it.*

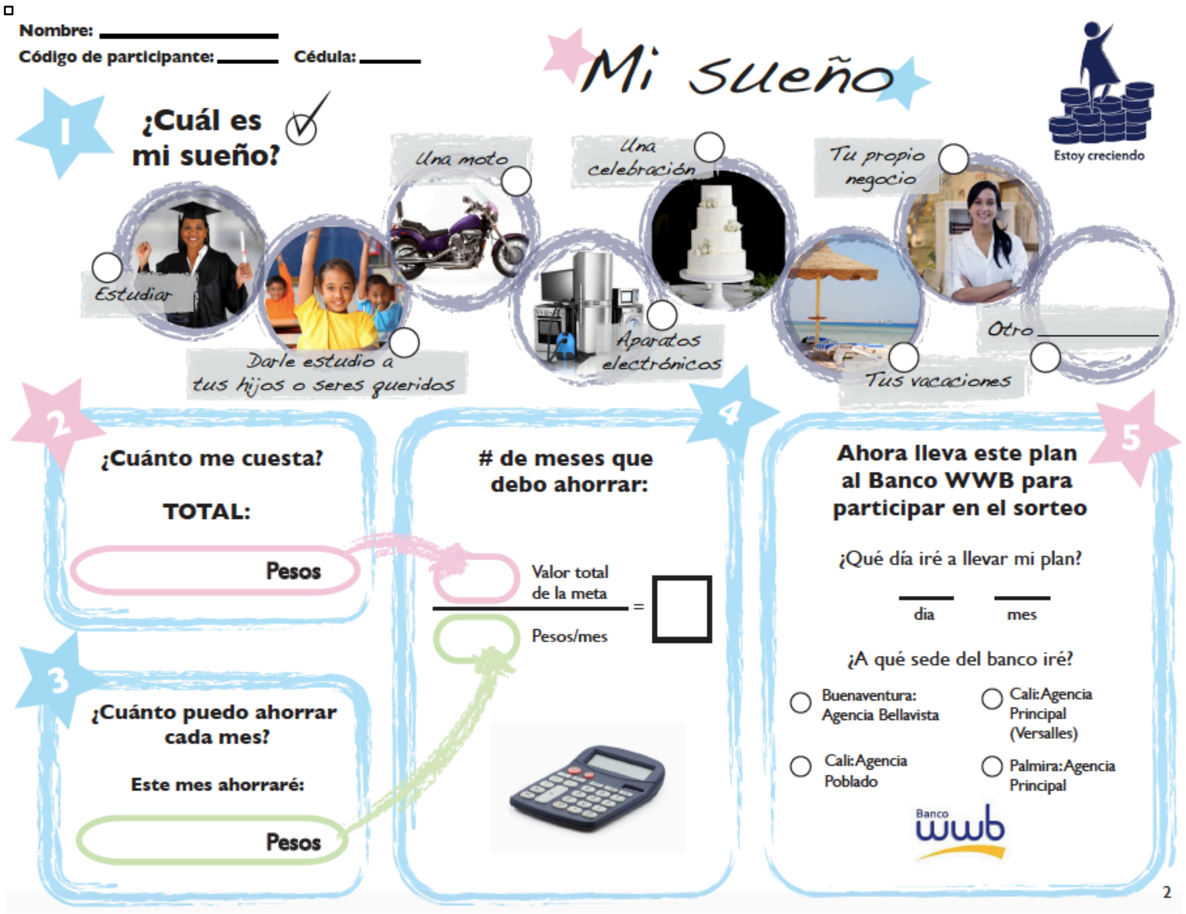

Figure S5. Example savings treatment lottery submission (goal-planning worksheet provided to financial treatment arms after 9-month survey)

F. References

1. Kim J, Ferrari G, Abramsky T, et al. Assessing the incremental effects of combining economic and health interventions: The IMAGE study in South Africa. *Bull World Health Organ*. 2009;87:824-832.
2. Ashraf N, Karlan D, Yin W. Female empowerment: Impact of a commitment savings product in the Philippines. *World Dev*. 2009;38(3):333-344.
3. Devos T, Spini D, Schwartz S. Conflicts among human values and trust in institutions. *Br J Soc Psychol.* 2002;41:481-494.
4. Adler NE, Epel ES, Castellazzo G, Ickovics JR. Relationship of subjective and objective social status with psychological and physiological functioning: Preliminary data in healthy white women. *Health Psychol.* 2000;19(6):586-592.
5. Bandura A. The explanatory and predictive scope of self-efficacy theory. *J Soc Clin Psychol.* 1986;4:359-373.
6. Kim JC, Watts CH, Hargreaves JR, et al. Understanding the impact of a microfinance-based intervention on women's empowerment and the reduction of intimate partner violence in South Africa. *Am J Public Health.* 2007;97(19):1794-1802.
7. Heatherton TF, Polivy J. Development and validation of a scale for measuring state self-esteem. *J Pers Soc Psychol.* 1991;60:895-910.
8. Brothers A, Chui H, Diehl M. Measuring future time perspective across adulthood: Development and evaluation of a short multidimensional questionnaire. *Gerontologist*. 2014;54:1075-1088.
9. Carstensen LL, Lang FR. Future orientation scale. Unpublished manuscript, Stanford University. 1996.
10. Pitt MM, Shahidur RK, Cartwright J. Empowering women with microfinance: Evidence from Bangladesh. *Econ Devel Cult Change*. 2006;54(4):791-831.
11. Cohen S, Kamarck T, Mermelstein R. A global measure of perceived stress. *J Health Soc Behav.* 1983;24(4):385-396.

12. Cohen S, Williamson GM. Perceived stress in a probability sample of the United States. In: Spacapan S, Oskamp S, eds. The Social Psychology of Health. Newbury Park, CA: Sage; 1988:31-67.

13. Losada A, Marquez-Gonzalez M, Garcia-Ortiz L, Gomez-Marcos MA, Fernandez-Fernandez V, Rodriguez-Sanchez E. Loneliness and mental health in a representative sample of community-dwelling Spanish older adults. *J Psychol*. 2012;143(3): 277-292.

14. Radloff LS. The CES-D Scale: A self-report depression scale for research in the general population. *Appl Psychol Meas.* 1977;1(3):385-401.

15. Beck AT, Epstein N, Brown G, Steer RA. An inventory for measuring clinical anxiety: Psychometric properties. *J Consult Clin Psychol*. 1988;56(6):893-897.

16. Magan I, Sanz J, Garcia-Vera MP. Psychometric properties of a Spanish version of the Beck Anxiety Inventory (BAI) in general population. *Span J Psychol*. 2008;11(2):626-640.

17. Basile KC, Hertz MF, Back SE. Intimate partner violence and sexual violence victimization assessment instruments for use in healthcare settings. Atlanta, GA: Centers for Disease Control and Prevention, National Center for Injury Prevention and Control; 2007.

18. DANE: Departamento Administrativo Nacional de Estadistica. Estratificacion social: Preguntas frecuentes. http://www.dane.gov.co/files/geoestadistica/Preguntas_frecuentes_estratificacion.pdf. Published 2013. Accessed June 16, 2017.
